# Lignosulfonate-Based Conducting Flexible Polymeric Membranes for Liquid Sensing Applications

**DOI:** 10.3390/ma14185331

**Published:** 2021-09-15

**Authors:** Sandra Magina, Alisa Rudnitskaya, Sílvia Soreto, Luís Cadillon Costa, Ana Barros-Timmons, Dmitry V. Evtuguin

**Affiliations:** 1 CICECO—Aveiro Institute of Materials and Department of Chemistry, University of Aveiro, 3810-193 Aveiro, Portugal; smagina@ua.pt (S.M.); anabarros@ua.pt (A.B.-T.); 2 CESAM and Department of Chemistry, University of Aveiro, 3810-193 Aveiro, Portugal; alisa@ua.pt; 3 I3N and Department of Physics, University of Aveiro, 3810-193 Aveiro, Portugal; silvia.soreto@ua.pt (S.S.); kady@ua.pt (L.C.C.)

**Keywords:** lignosulfonate, potentiometric sensors, carbon nanotubes, conducting polymer, electrical conductivity, transition metals, graphene, ionic liquid

## Abstract

In this study, lignosulfonate (LS) from the acid sulfite pulping of eucalypt wood was used to synthesize LS-based polyurethanes (PUs) doped with multiwalled carbon nanotubes (MWCNTs) within the range of 0.1–1.4% *w*/*w*, yielding a unique conducting copolymer composite, which was employed as a sensitive material for all-solid-state potentiometric chemical sensors. LS-based PUs doped with 1.0% *w*/*w* MWCNTs exhibited relevant electrical conductivity suitable for sensor applications. The LS-based potentiometric sensor displayed a near-Nernstian or super-Nernstian response to a wide range of transition metals, including Cu(II), Zn(II), Cd(II), Cr(III), Cr(VI), Hg(II), and Ag(I) at pH 7 and Cr(VI) at pH 2. It also exhibited a redox response to the Fe(II)/(III) redox pair at pH 2. Unlike other lignin-based potentiometric sensors in similar composite materials, this LS-based flexible polymeric membrane did not show irreversible complexation with Hg(II). Only a weak response toward ionic liquids, [C_2_mim]Cl and ChCl, was registered. Unlike LS-based composites comprising MWCNTs, those doped with graphene oxide (GO), reduced GO (rGO), and graphite (Gr) did not reveal the same electrical conductivity, even with loads up to 10% (*w*/*w*), in the polymer composite. This fact is associated, at least partially, with the different filler dispersion abilities within the polymeric matrix.

## 1. Introduction

Due to an increase in industrial and human activities, the presence of heavy metal salts such as lead (Pb), mercury (Hg), cadmium (Cd), chromium (Cr), zinc (Zn), and copper (Cu), among others, in wastewater has led to an increasing accumulation of these chemicals in the environment. Heavy metal ions are non-degradable, toxic, and harmful to aquatic life and can cause many health problems as they accumulate in the human body through the food chain. Considering environmental and health concerns, their removal from wastewaters is imperative [1]. Therefore, their detection in aqueous systems is crucial. Electrochemical sensors, such as potentiometric, amperometric, and conductometric sensors, are particularly interesting for environmental water monitoring as they are suitable for the determination of chemical species such as heavy metals, among others. They are user-friendly, well-suited for miniaturization, have short response times, a wide dynamic range, low energy consumption, low cost, ease of preparation, good sensitivity, and high selectivity [2,3,4]. However, some problems still need to be addressed, such as poor reproducibility and stability and difficulties in analyzing complex matrices and real samples, and some ions may act as ligands and poison the electrode (for instance, mercury) [4,5]. All-solid-state potentiometric sensors are promising sensors for in situ water analysis [6]. They have been employed in numerous studies for the determination of metal ions in water and biological samples (such as sweat) [3,4,7] and drug molecules in various biological samples (such as blood serum and urine) [8]. Furthermore, the risk of toxic and other harmful effects related to the use of other chemical species, such as, for instance, ionic liquids (ILs) and their contribution to environmental pollution through release via wastewater effluents [9], enhances the need for the development of sensors suitable for the detection of a wider range of chemicals.

Conducting polymers (CPs) are an important class of organic multifunctional materials that exhibit specific physical and electrical properties. The possibility of fine-tuning their optical and conducting properties makes them promising candidates for a wide range of applications, including the fields of energy [10,11,12], electronics [13,14], catalysis [15], electromagnetic interference shielding [16,17,18], biomedicine [7,8], and sensors [3,7,8,12,19,20,21,22]. CPs usually exhibit relatively low conductivity (~10^−8^–10^−3^ S·m^−1^) in their pure state; nonetheless, this property can be effectively enhanced to ~100–10^6^ S·m^−1^ either by chemical or electrochemical doping in the case of intrinsic CPs or by physical mixing with other electro-conducting species such as metal or metal oxide nanoparticles, metal-organic frameworks (MOFs), and carbon-based nanomaterials, among others [19,20,23]. According to previous results, both phenomena have been observed in lignin-based polyurethanes mixed with MWCNTs [24].

Carbon-based materials, such as carbon nanotubes (CNTs), graphene, carbon dots, and porous carbon, have attracted scientific interest worldwide since these nanofillers exhibit enhanced conductivity, high chemical stability, mechanical strength, and large surface areas. Therefore, numerous studies have focused on the preparation of CP composites using these carbon-based materials as nanofillers in order to improve their electrical and mechanical properties but also to allow the production of small, lightweight, and cost-effective composites for a variety of application fields, including electronics, energy, automotive, and aerospace industries and sensors [19,23,25,26].

Graphite is one of the three naturally occurring allotropes of carbon and is a crystalline material consisting of 2D-layered sp^2^-bonded carbon atoms arranged in a planar hexagonal structure. Graphene consists of one atomic layer of graphite and exhibits extremely high mechanical properties (1.1 TPa modulus), electrical conductivity (10^8^ S·m^−1^), thermal conductivity (5000 W·mK^−1^), and optical properties (98% transmittance) [26]. GO derives from the oxidation of pristine graphene and, hence, is a 2D atomically thin layer of hexagonal sp^2^-bonded carbon atoms that comprises oxygen-based functional groups such as hydroxyl (-OH), alkoxy (C-O-C), and carboxylic (-COOH) acids, among others. These oxygen functionalities disrupt the sp^2^-conjugated backbone, causing a reduction of its electrical conductivity. To restore its electrical conductivity, some oxygen functional groups in GO can be partially reduced, yielding rGO [27]. CNTs are 1D conductive fillers due to their high length-to-diameter ratio, consisting of a hexagonal arrangement of sp^2^-hybridized carbon atoms, which may be shaped by rolling up a single sheet of graphene (single-walled carbon nanotubes, SWCNTs) or by rolling up multiple sheets of graphene (multiwalled carbon nanotubes, MWCNTs). CNTs present impressive electrical conductivities (10^7^–10^8^ S·m^−1^) and mechanical properties (1 TPa modulus, 100 GPa strength) [26].

Due to the continued depletion of finite fossil resources and the necessity to reduce the dependence on this type of resource and their environmental impacts, alternative and sustainable resources have been assessed as feedstock replacements, namely, biomass. Biorefinery and circular economy concepts have become imperative in the industrial sector, e.g., the pulp and paper industry. Thus, in order to improve the economic sustainability of pulp mills, the valorization of the ensuing by-products has been directed towards new added-value products to expand their economic profits. In particular, industrial (technical) lignin has attracted worldwide attention as it is an abundant biodegradable and renewable resource for the production of biofuels, chemicals, and polymeric materials, with the advantage that it does not compete with food stocks. Lignins have potential as adsorbents for removing metals from water due to their acid sites, namely, carboxylic and phenolic groups [28,29,30], though the phenolic groups exhibit a higher affinity for metal ions than the carboxylic ones [28]. In addition, other functional groups, such as nitrogen- and sulfur-containing groups that may be present in the lignin structure, can also bind heavy metal ions [29,30]. In fact, lignin modification with nitrogen- and sulfur-containing functional groups has been used in the design and development of advanced adsorbents [29,30,31,32]. Among different technical lignins, lignosulfonate (LS) is the only abundant lignin source on the market that can be considered potentially interesting for the production of conducting composites. Being soluble in water and containing ionogenic (sulphonic) groups, LS is an attractive candidate for use in conductive polymer matrices for sensor applications. In previous studies, novel lignin-based sensing polymeric membranes have been developed through covalent immobilization of the lignin inside a polymer matrix [5,24,33,34]. Furthermore, the production of lignin-based PU membranes through co-polymerization of different technical lignins, such as kraft lignin, LS, and organosolv lignin, with toluene diisocyanate-terminated poly(propylene glycol) has been reported [33]. Moreover, lignin-based PUs doped with MWCNTs allowed the increase of composite electrical conductivity with a prospective application as ion-selective membranes for potentiometric chemical sensors [5,34]. However, the ensuing lignin-based sensors displayed very low or no sensitivity to all alkali, alkali-earth, and most transition metal cations ions. At the same time, the sensor properties were strongly dependent on the lignin origin. Thus, some concomitants of polyphenolic origin in kraft lignin imparted to this material sensitivity and selectivity to Cu(II) [5]. Potentiometric sensors highly sensitive to Cr (VI) at pH 2 were obtained with LS and organosolv lignin [34]. However, with regards to LS, no systematic studies have been carried out on the effect of different types and loads of carbon-based fillers on the conducting performance of CPs.

In this work, we prepare LS-based PU composite membranes sensors doped with MWCNTs using the same synthesis methodology as in previous studies [5,24,33,34]. The main goal is to assess the viability of using LS from the spent liquor of the acidic sulfite pulping of eucalypt wood for potentiometric sensor applications. The conductive and sensory properties of the CP based on LS are compared with those of the CP based on eucalyptus kraft and kraft LignoBoost^®^ lignin, organosolv lignin, and lignosulphonate obtained under different cooking conditions [5,34]. In addition, various carbon nanofillers such as graphene oxide (GO), reduced graphene oxide (rGO), and graphite (Gr) are used to dope LS-based PU composite for comparison.

## 2. Materials and Methods

### 2.1. Materials and Reagents

Lignosulfonates (LS) from the industrial magnesium-based acidic sulfite pulping of *Eucalyptus globulus* wood were supplied by Caima Company (Constância, Portugal) and purified by dialysis against distilled water for 24 h. Purified LS contains 17.1% wt.% of HSO_3_ groups and 2.4 wt.% of phenolic hydroxyl groups [35].

Poly(propylene glycol)-toluene diisocyanate copolymer (PPGDI, with average Mn ~2300 g·mol^−1^, DP ~34, and isocyanate content ~3.6 wt.%) and dibutyltin dilaurate (DBTDL) were purchased from Sigma-Aldrich (Madrid, Spain). Multiwall carbon nanotubes (MWCNTs) Nanocyl-3150 (purity >95%, length 1–5 µm, and diameter 5–19 nm) were supplied from Nanocyl, S.A. (Sambreville, Belgium). Graphene oxide (GO) and reduced graphene oxide (rGO) were supplied by Graphenea (San Sebastián, Spain). Graphite was supplied by Graphite Technologies, Lda (Oliveira de Azeméis, Portugal). All solvents and other reagents, namely, 2-amino-2-(hydroxymethyl)-1,3-propanediol (Tris), potassium dichromate, chromium(III) chloride hexahydrate, zinc(II) chloride, lead(II) nitrate, ammonia, sodium nitrate, cadmium(II) nitrate, copper(II) chloride, silver(I) nitrate, potassium ferrocyanide(II), potassium ferricyanide(III), mercury(II) chloride, 1-ethyl-3-methylimidazolium chloride, and choline chloride were of analytical grade and were purchased from either Acros or Sigma-Aldrich Chem. Comp (Madrid, Spain).

Polyaniline (PANI)-modified screen-printed electrodes (SPEs) with carbon working and auxiliary electrodes and a silver reference electrode were supplied by Metrohm DropSens (Oviedo, Spain). All solutions for the potentiometric measurements were prepared using ultrapure water (18 mΩ·cm^−1^).

### 2.2. LS-Based PU Polymer Synthesis

The polycondensation reaction of purified LS with isocyanate was carried out as described previously [5,24,33,34,36], with some adjustments. For all syntheses, the amounts of LS and PPGDI were chosen in order to obtain an NCO/OH ratio of 1.5. Firstly, LS was ground using an agate mortar and pestle and then mixed with MWCNTs (without grinding). In a typical trial, LS powder (500 mg) or a mixture of LS powder with a certain proportion of MWCNT (0.1, 0.2, 0.5, 0.8, 1.0, and 1.4% *w*/*w* in relation to the total mass of the mixture, i.e., LS and PPGDI) was placed in a 25 mL jacketed glass reactor equipped with an overhead mechanical stirrer and a heated circulating water bath. Then, PPGDI (4 mL, *d* 1.05 g·cm^−3^) was added, and the mixture was stirred for 45–60 min at 60 °C under a nitrogen atmosphere to obtain a homogeneous viscous mixture. Then, DBTDL (ca. 2% *w*/*w* in relation to the PPGDI) was added. The homogeneous mixture was stirred for a further 5–10 min until it started to thicken. At this point, the mixture was removed from the reactor and poured into a flat PTFE mold. The films were cured for 4 h at 60 °C. The LS-based PU films obtained were submitted to chemical, thermo-mechanical, and electrical characterization. The sensors were prepared by placing a thin layer of the LS-based polymer on the working electrode of SPE. The sensors were cured for 4 days at room temperature. At least three parallel sensors of the same composition were prepared.

### 2.3. Polymer Characterization

LS-based polymer films were characterized by Fourier transform mid-infrared spectroscopy (FT-MIR) using an FTIR System Spectrum BX instrument (PerkinElmer, Waltham, MA, USA), coupled with a universal ATR sampling accessory, in absorbance mode from 4000 to 500 cm^−1^ with a 4 cm^−1^ resolution. Samples were analyzed as such, 128 scans were averaged, and all spectra were baseline-corrected for further analysis.

Dynamic mechanical analysis (DMA) of the polymer films was carried out using a Tritec 2000 DMA instrument (Triton Technology, Leicestershire, UK). A rectangular piece of LS-based polymer or corresponding composites with dimensions of 30 × 5 mm^2^ was mounted in tension geometry and then submitted to a temperature scan using a constant heating rate of 2 °C·min^−1^, from −100 °C up to 60 °C ((before starting the run, an initial static force was applied (1 N) to guarantee that the sample remains under a net tensile force). The displacement was 0.010 mm, and the frequency of deformation (oscillating frequency) was alternated between 1 and 10 Hz as the temperature was increased. The glass transition temperature, *T*_g_, of the polymers was determined from the maximum value of the peak in tan δ, the damping factor, which is defined as the ratio of loss to storage modulus.

Thermogravimetric analysis (TGA) of the samples was carried out using a Setsys Evolution 1750 TGA-DSC thermogravimetric analyzer (Setaram, Caluire, France) equipped with a DSC plate rod accessory. Samples were analyzed from room temperature up to 800 °C at a heating rate of 10 °C·min^−1^ under nitrogen gas and a flow rate of 200 mL·min^−1^ using an alumina crucible.

SEM images of lignin and lignin-based polyurethanes were recorded using a Hitachi S-4100 microscope (Hitachi, Tokyo, Japan) on the gold-coated samples, applying an acceleration voltage of 5 kV.

DC electrical conductivity was measured at temperatures between −110 and 100 °C using a 617 Keithley electrometer (Keithley Instruments GmbH, Munich, Germany). Electrical contacts were made by painting LS-based polymer or corresponding composite films on both sides with silver paste, simulating a parallel plate capacitor with a surface area of about 1 cm^2^ and 1 mm distance between electrodes.

Dielectric measurements for frequencies between 100 and 1 MHz were carried out using an Agilent 4294A precision impedance analyzer (Agilent, Santa Clara, CA, USA) at temperatures between −82 and 108 °C under a helium atmosphere. Electrical contacts were made by placing the LS-based polymer or corresponding composite films between two electrodes, simulating a parallel plate capacitor with a surface area of about 1 cm^2^ and distance between electrodes of 1 mm.

### 2.4. Potentiometric Measurements

Potentiometric chemical sensors were prepared by depositing a thin layer of LS-based PU doped with 1% *w*/*w* MWCNTs (before curing) on the surface of PANI-SPE. Then the polymer was cured at room temperature in a desiccator for 4 days before use.

Electrochemical measurements were carried out in the following galvanic cell:

Ag|AgCl, KClsat|sample|polymer membrane|PANI|carbon.

Electromotive force values, *E_mf_*, were measured vs. a Ag/AgCl reference electrode with a precision of 0.1 mV using a custom-made multichannel voltmeter, with high input impedance, connected to the PC for data acquisition and processing. Calibration measurements were made in the solutions of zinc nitrate, cadmium nitrate, lead nitrate, copper(II) chloride, mercury(II) chloride, silver nitrate, chromium(III) chloride, potassium dichromate, potassium ferrocyanide, and potassium ferricyanide in the concentration range 1.0 × 10^−7^–1.0 × 10^−2^ M. Tris buffer solution with a concentration of 1 mM and pH 7, adjusted by addition of hydrochloric acid, was used as a supporting electrolyte. Redox response was studied in the solutions of two redox pairs, Cr(III)/Cr(VI) and Fe(CN)_6_^3−/4−^, at pH 2 on the background of 0.01 M HCl, and at pH 7 on the background of 1 mM Tris buffer solution. Total concentration was 1 mM for both pairs, with the ratio of oxidized to reduced form varying from 0.01 to 100. The parameters of the Nernst equation, i.e., the slope of electrode function and standard potential, were calculated using linear regression and averaged over replicated calibration runs for each ion.

Sensor selectivity was estimated using the matched potential method (MPM) at pH 7 on the background of Tris buffer solution. At least 3 replicated measurements were run for each ion.

## 3. Results and Discussion

LS-based flexible copolymers were obtained by the reaction of LS (*M_w_* = 4130 Da) as a macromonomer bearing hydroxyl groups with PPDGI (*M_n_* = 2300 g·mol^−1^) as a co-macromonomer comprising isocyanate groups and also acting as the solvent for LS, maintaining the recommended NCO/OH ratio of 1.5 [5,24,33,34]. The conducting carbon nanofillers, in particular MWCNTs, were introduced jointly with lignin before the synthesis, taking into account the excellent dispersant capability of the former [33,34]. The obtained materials were characterized and analyzed for their electrical conductivity and sensor properties. 

### 3.1. Characterization of LS-Based PUs

**Figure 1:**
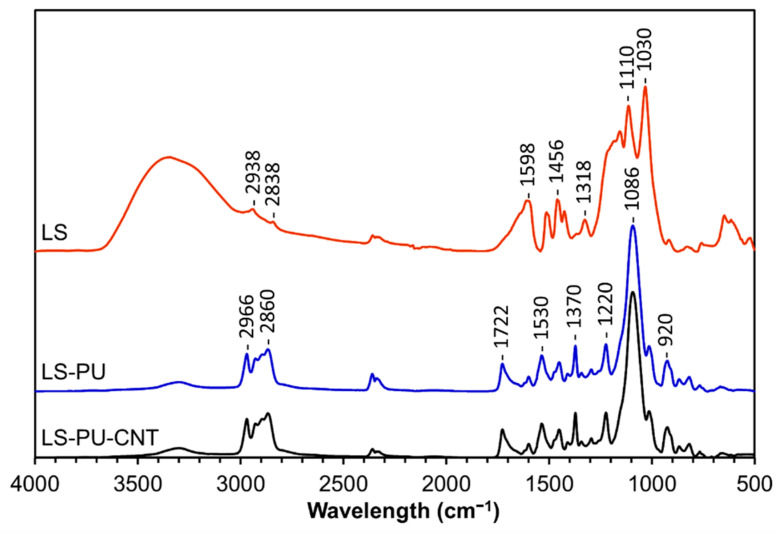
FTIR-ATR spectra of LS, LS-based PU (LS-PU), and LS-based PU doped with 1% *w*/*w* MWCNTs (LS-PU-CNT).

The reaction of LS and PPDGI with the formation of PU was confirmed by FTIR spectroscopy. Figure 1 shows the FTIR spectra of purified LS and LS-based PU without (LS-PU) or with 1% *w*/*w* MWCNTs (LS-PU-CNT) taken as an example. The formation of PU is confirmed by the absence of the band at ca. 2270 cm^−1^, assigned to the isocyanate group (-NCO) in PPDGI, and the appearance of signals characteristic for urethane (–O–(C=O)–NH–) moieties at 1722, 1370 and 1220 cm^−1^, assigned to C=O, O–CO, and C-N stretching, respectively [33,37,38]. Furthermore, the significant decrease in the relative intensity of the OH band around 3350 cm^−1^ in the spectra of both LS-based PUs indicates that a relevant amount of LS hydroxyl groups was consumed during the polymerization reaction with the isocyanate groups. The characteristic aromatic bands of LS at 1598, 1510, and 1425 cm^−1^ [39,40,41,42,43,44,45,46] decreased substantially in the LS-based PU due to the relatively low LS content (ca. 20% *w*/*w*). The most abundant signals in the LS-based PU spectrum, at 1086 and 2860 cm^−1^, belong to C–O and CH_2_ vibrations, respectively, in polyether bridges connecting lignin macromolecules (for more details, see Table S1 in the Supplementary Materials). Although no significant spectral differences were found between LS-PU and LS-PU-CNT, the slightly greater abundance of free hydroxyl moieties at ca. 3350 cm^−1^ in LS-PU-CNT can be explained by the strong interaction of MWCNTs and lignin [33,34], thus hindering, to a certain degree, their reaction with PPDGI.

**Figure 2:**
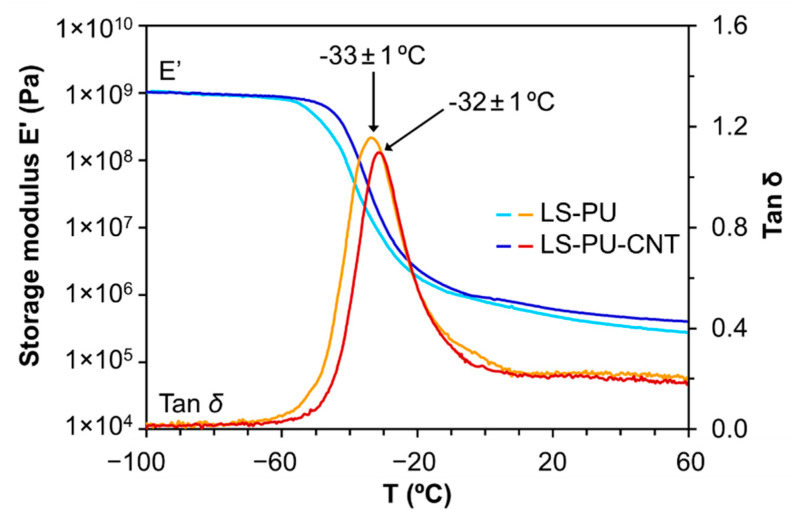
DMA profiles of LS, LS-based PU (LS-PU), and LS-based PU doped with 1% *w*/*w* MWCNTs (LS-PU-CNT).

The glass transition temperature (*T*_g_) of LS-based PUs was assessed by DMA. Both LS-based PUs, undoped and doped with 1% *w*/*w* MWCNTs, exhibited similar low *T*_g_ values, −33 ± 1 °C and −32 ± 1 °C, respectively, as depicted in Figure 2. This relaxation peak relates to the soft domains of the polymeric composites [38]. The addition of MWCNTs (1% *w*/*w*) did not affect the viscoelastic properties of the polymer. The negative values of *T*_g_ indicate that these materials can be used for the fabrication of self-plasticizing membranes for potentiometric chemical sensors.

**Figure 3:**
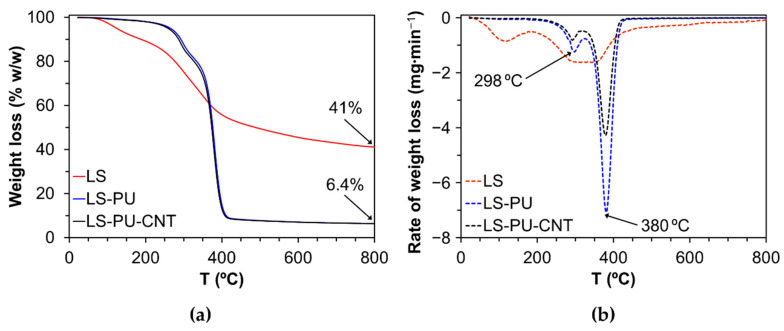
TGA curves of LS, LS-based PU (LS-PU), and LS-based PU doped with 1% *w*/*w* MWCNTs (LS-PU-CNT): (**a**) weight loss under inert N_2_ gas flow and (**b**) derivative of the weight loss.

Typical TGA curves of LS and LS-based PUs, undoped and doped with 1% *w*/*w* of MWCNTs, are depicted in Figure 3. LS revealed less thermal stability than the corresponding PU. The weight loss, with a maximum loss at around 120 °C, is related to moisture release followed by the degradation of functional groups and the release of low molecular mass products at temperatures as high as 200 °C (e.g., SO_2_ from sulphonic groups) [47,48]. Char formation begins at ca. 400 °C. Both LS-based PU polymers (undoped and doped with MWCNTs) exhibited improved thermal stability compared to LS since the degradation starts at ca. 275 °C (Figure 3). Additionally, LS-based PU samples exhibited a two-stage degradation process, which corresponds to the thermal decomposition of the hard and soft segments, respectively [38,49]. Hard segments include the thermally weaker urethane moieties, where the maximum rate of weight loss occurred at 298 °C. Soft segments are essentially associated with the polyether moieties from the PPGDI co-macromonomer, where the maximum rate of weight loss occurred at 380 °C. The thermal degradation profile of both LS-based PU polymers (undoped and doped with MWCNTs) is similar since the amount of MWCNTs (1% *w*/*w*) is too low to cause a significant change in the thermal behavior of the doped LS-based PU, at least under an inert gas atmosphere.

### 3.2. DC and AC Electrical Conductivity of LS-Based PU Polymer Doped with MWCNTs

**Figure 4:**
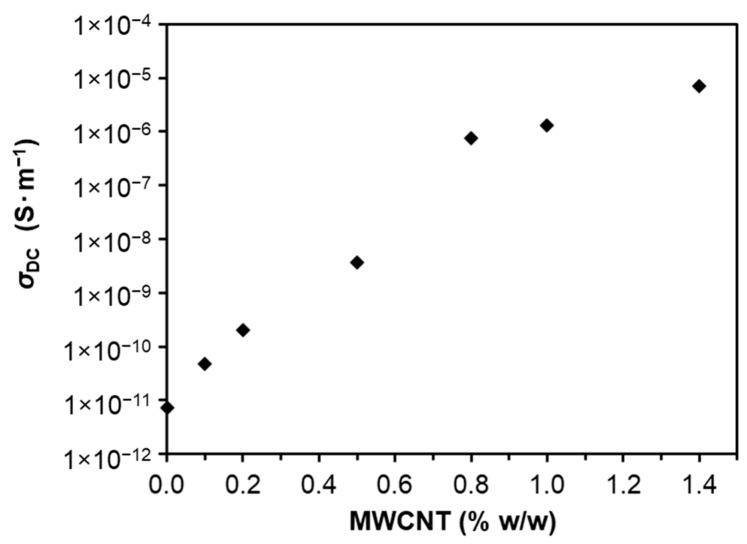
DC electrical conductivity, *σ*_DC_, at room temperature as a function of MWCNT concentration (0%, 0.1%, 0.2%, 0.5%, 0.8%, 1%, 1.2%, and 1.4% *w*/*w*) in LS-based PU.

The DC electrical conductivity of undoped LS-based PU polymer and the various LS-based PU polymer composites doped with variable amounts of MWCNTs was measured at room temperature, as depicted in Figure 4. Noteworthy is the fact that neat LS-based PU polymer is not a completely insulating material, exhibiting low electrical conductivity mostly due to the presence of LS in the polymeric matrix. This might be due to the mixed type electrical conductivity associated with the presence of π-conjugated aromatic moieties and of ionogenic functional groups such as carboxyl and sulphonic groups in lignosulfonates [50]. The incorporation of MWCNTs resulted in an increase in the electrical conductivity of PU-based LS composites in the order of magnitude 10^6^, from 7.2 × 10^−12^ in undoped polymer to 7.1 × 10^−6^ S·m^−1^ in polymer doped with 1.4% *w*/*w* MWCNTs (Figure 4). However, the observed conductivity curve as a function of MWCNT content in the composite did not fit the percolation theory model generally applied to composites doped with carbon nanotubes [25,51,52].

Unlike other PU-based composites produced from kraft or organosolv lignins [34], which show a sharp percolation threshold at low concentrations of MWCNTs (0.2–0.7% *w*/*w*), the LS-based PU did not reveal that feature, and the increase in conductivity with the addition of MWCNTs was not exponential (Figure 4). The plausible explanation for this conductivity behavior could be the greater aggregation of polar LS in the PPDGI copolymer in comparison with the non-polar kraft and organosolv lignins. LS also has a molecular weight 2–3 times greater than kraft lignin, which makes it difficult to dissolve. In fact, the TEM image of a thick film of LS-based PU doped with 1% MWCNT showed large LS aggregates (Figure S1, Supplementary Materials).

**Figure 5:**
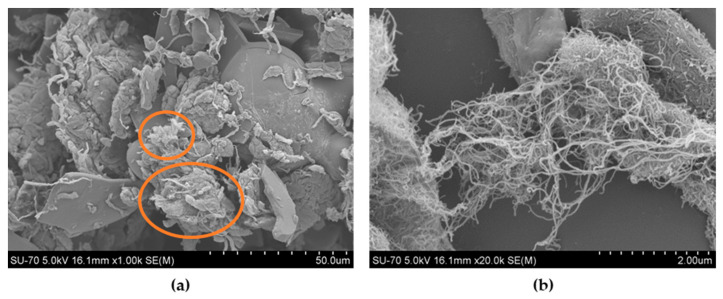
SEM image of a mixture of LS with 1% *w*/*w* MWCNTs (**a**) with an expanded image of MWCNT bundles bound to lignin particles (**b**). Examples of large MWCNT agglomerates are depicted by circles.

Since lignin, including LS, is a dispersant of MWCNTs, its dissolution in co-macromonomer (PPDGI) is a crucial factor affecting the percolation phenomena that could compromise the formation of a continuous conductive network within the composite matrix. In fact, MWCNTs bind readily to LS, as can be seen from SEM images of the LS powder mixed with 1% *w*/*w* MWCNTs (before adding the co-macromonomer PPGDI), thus forming bundles with a highly entangled structure (Figure 5). The inhomogeneous distribution of LS in the copolymer (final composite after curing) could negatively affect the long-range connectivity between the bound conductive elements within the LS-based polymeric network structure, thus reducing the electrical conductivity of the ensuing composite. In addition, the composite doped with MWCNTs revealed a more porous structure due to the more intensive bubble formation during the synthesis, which also negatively affected percolation conductivity (Figure S2, Supplementary Materials). These bubbles are trapped within the composite matrix, disrupting the conductivity path, i.e., the long-range connectivity between the conductive elements, hindering and even interrupting conductivity, which may also contribute to explaining why the composites do not display a common percolation behavior.

**Figure 6:**
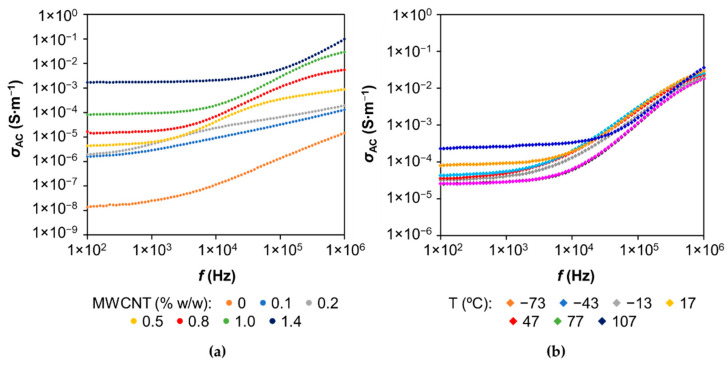
Frequency dependence of AC electrical conductivity, *σ*_AC_, for LS-based PU polymer doped with different amounts of MWCNT at 77 °C (**a**) and with 1% (*w*/*w*) MWCNT at different temperatures (**b**).

Alternating current conductivity, $\sigma_{\mathrm{AC}}$, of LS-based PU polymers undoped and doped with different amounts of MWCNTs and the temperature effect on *σ*_AC_ variation as a function of frequency for LS-based PU polymer composites doped with 1% *w*/*w* MWCNTs are shown in Figure 6. At first glance, the frequency dependence of AC conductivity can be divided into two discrete domains. At low frequencies (<1 kHz), AC conductivity is nearly constant and independent of the frequency, and its value comes close to the value of DC conductivity. However, in some cases, a very small slope is noticeable, especially for undoped LS-based PUs and LS-based PUs doped with 0.1% *w*/*w* MWCNTs. The frequency region of constant conductivity extends to higher frequencies with increasing concentrations of MWCNTs. Above a certain frequency, AC conductivity increases with increasing frequency. Hence, at higher frequencies, total AC conductivity, $\sigma_{\mathrm{AC}}\left(\omega \right)$, is frequency-dependent, and its increase obeys a power law (known as the Jonscher universal power law [53]) given by Equation (1):

(1) $$\sigma_{\mathrm{AC}}(\omega )=\sigma_{\mathrm{DC}}+A\omega^{s}.$$

where *ω* is the angular frequency (*ω* = 2πf, where *f* is the frequency), $\sigma_{\mathrm{DC}}$ is the independent frequency (DC) conductivity at *ω*→0, *A* is a constant dependent on temperature *T*, and *s* is an exponent dependent on both frequency and temperature, with values in the range 0–1. This power law is characteristic of disordered materials in which the conductivity is due to the hopping of charge carriers between localized states [54]. It is also presumed that *A* and *s* values are interrelated and have a mechanistic origin in terms of underlying disordered microscopic structures [55]. In fact, within the range of −13 to 107 °C, corresponding to the viscoelasticity interval, the straight-line correlation between Log *A* and *s* has been observed (Figure S3, Supplementary Materials). Within the same temperature range, almost constant log *A*/*s* values (nearly 20) were registered while analyzing the log *A*/*s* vs. *T* dependency from the universal Jonscher equation ($\sigma_{\mathrm{AC}}=A\omega^{s}$). Similar behavior was observed previously with epoxy polymeric formulations doped with MWCNTs and assigned to the unchanged composite morphology of the polymer-MWCNT network when charge carriers migration was affected exclusively by conformational rearrangements of disordered microscopic structures [55]. Additionally, at lower frequencies, AC conductivity is temperature-dependent, evidencing that the conductivity is a thermally activated process. The activation energy was assessed from the known Arrhenius equation while analyzing the plot ln $\sigma_{\mathrm{AC}}$ vs. *1*/*T* in the temperature range of −13 to 107 °C [5]. The relatively low activation energy for $\sigma_{\mathrm{AC}}$ at *ω*→0 of the PU composite containing 1% MWCNTs (0.13 eV), when compared with that of the undoped PU composite (0.59 eV), clearly indicated the significant interaction between MWCNT and LS. This feature was similar to those observed previously with PUs doped with MWCNTs using kraft and organosolv lignins synthesized under the same conditions [5,34]. This fact supports the idea about similar conductivity mechanisms in all these lignin-based PUs doped with MWCNTs [5,34] despite no clear percolation threshold being observed with LS (Figure 4).

**Figure 7:**
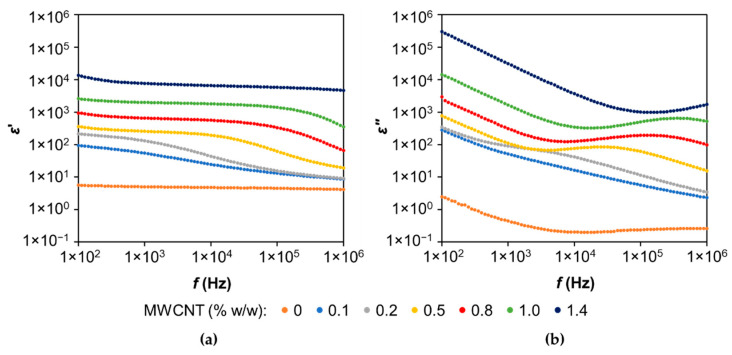
Real *ε*′ (**a**) and imaginary *ε*″ (**b**) parts of complex permittivity, ε*(*f*) = *ε*′(*f*) − *iε*″(*f*) as a function of frequency, at *T* = 77 °C for LS-based polymer undoped and doped with different amounts of MWCNTs.

The real and imaginary parts of the dielectric permittivity, *ε*′ and *ε*″, respectively, of LS-based PUs undoped and doped with different amounts of MWCNTs are presented in Figure 7 in the frequency range of 100 Hz to 1 MHz. Both real and imaginary parts of dielectric permittivity decrease with increasing frequency, especially the imaginary part, which is highly frequency-dependent. This behavior is probably due to the interfacial polarization effect on the polymeric composites, attributed to the accumulation of polarized charges from the difference in conductivities and permittivities of the constituents of the LS-based PU polymer composite (i.e., the polymer matrix and the CNTs) [56]. Additionally, at the same frequency, both *ε*′ and *ε*″ increase with the increase in the amount of MWCNTs, which is attributed to an increase in the density of the MWCNT network [56]. Another remark from the *ε*″ plot (Figure 7b) is that a relaxation process (in the shape of a broad peak) appears at higher frequencies and higher MWCNT contents (though a slight peak for the composite with 0.2% *w*/*w* MWCNTs is also observed). This relaxation process moves towards higher frequencies in accordance with an increase in MWCNT content in the composite. The peak of the highest concentration (1.4% *w*/*w* MWCNTs) is not visible since it falls outside the frequency window range. Further experiments are still necessary to fully understand the dielectric relaxation mechanisms of these composites.

Carbon nanotubes are considered to be the most promising reinforcement fillers used to improve the mechanical, electrical, and thermal properties of polymers [25]. In this particular study, when only small amounts of MWCNTs were used in the polymer matrix, electrical conductivity improvement was achieved, thus making these composites suitable for sensor applications. The synthesis of an LS-based PU composite doped with 1.4% *w*/*w* MWCNTs was a challenge since the mixture was very viscous, and it was very difficult to prepare the films by molding, which led to composites with highly irregular surfaces. Therefore, and considering the conductivity results, the LS-based PU composite containing 1% *w*/*w* MWCNTs was the formulation chosen for potential application as a potentiometric sensor.

### 3.3. Sensor Properties of the LS-Based PU Polymer Membrane Composite Doped with 1% w/w MWCNTs

**Figure 8:**
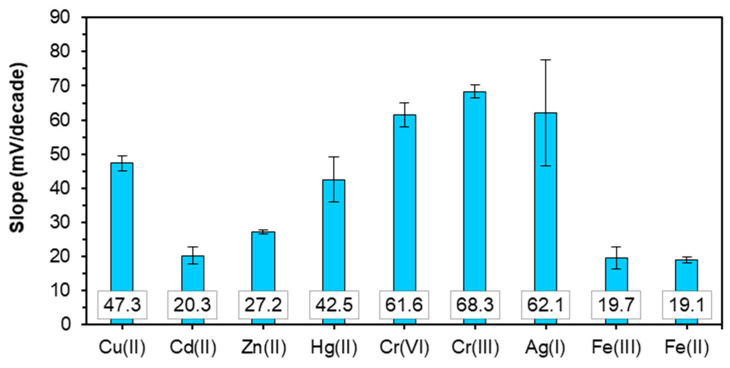
Slopes of the electrode function of the LS-based PU sensor doped with 1% *w*/*w* MWCNTs at pH 7 (mean values of at least three calibrations with their respective standard deviations).

**Figure 9:**
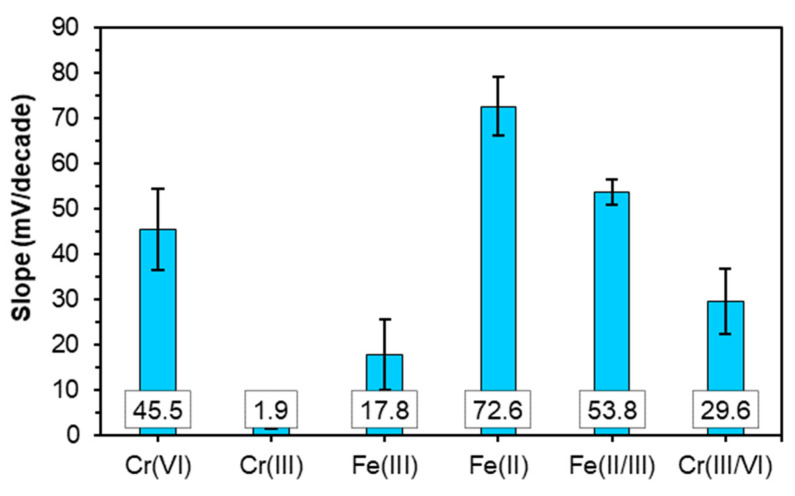
Slopes of the electrode function of the LS-based PU sensor doped with 1% *w*/*w* MWCNTs at pH 2 (mean values of at least three calibrations with their respective standard deviations).

Potentiometric measurements for a sensitivity assessment of an LS-based PU composite membrane sensor doped with 1% *w*/*w* MWCNTs were carried out using 1 mM Tris buffer solution at pH 7. The measurements revealed no response to Na^+^, NH^4+^, and Pb(II) but had a response to Cu(II), Cd(II), Zn(II), Cr(VI), Cr(III), and Ag(I) (Figure 8). Response slopes towards studied ions were calculated based on the Nernst equation (Equation (2)) at a working temperature of 25 °C:

(2) $$E=E^{0}\pm \frac{RT}{z_{i}F}loga_{i}.$$

where *E* is measured potential (EMF of the electrochemical cell), *E*^0^ is standard potential, *R* is the gas constant, *T* is the temperature in K, *F* is the Faraday constant, *a_i_* is the activity of the studied cation, and *z_i_* is the charge of the ion. A concentration variation by a factor of 10 should give rise to a potential variation of ca. 59 mV for single-charged ions, but, for double-charged ions, it should be around 30 mV, and, in the case of triple charged ions, the slope value should be around 20 mV. Response slopes of the LS-based PU doped with a 1% *w*/*w* MWCNT electrode towards different ions at pH 7 and 2 are depicted in Figure 8 and Figure 9, respectively, and the detection limits and linear ranges are presented in Table S2 (Supplementary Materials). The detection limit of the sensor was determined from the intersection of two extrapolated segments of the calibration plots according to IUPAC recommendations [57].

The sensor exhibits a low response to Cd(II) (with a slope of 20.3 ± 2.5 mV) and a near-Nernstian response towards Zn(II) (with a slope of 27.2 ± 0.7 mV) and Ag(I) (with a slope of 62.1 ± 15.5 mV). For other cations such as Cu(II) and Hg(II), the super-Nernstian responses were obtained at pH 7 (Figure S4, Supplementary Materials). Responses to mercury and silver ions are associated with high standard deviations, as after the first calibration measurement in the solutions of these ions, the slopes of the electrode decreased. Furthermore, some decrease of the sensor response slopes was observed in the solutions of copper and zinc after sensor exposure to mercury and silver, indicating an eventual partially irreversible interaction of the polymer membrane with these ions. At the same time, no drastic, irreversible complexation with Hg(II) ions was detected, compared with other lignin-based sensor membranes of the same polymer composite [5,33,34].

Considering that at pH 7, the predominant species of Cr(III) is Cr(OH)_2_^+^, the sensor response to this ion is slightly above the theoretical one, i.e., 68 mV/pX instead of the expected 59 mV/pX. No response to Cr(III) was observed at pH 2, which is in agreement with the literature data of Cr(III) adsorption by different sorbents and is attributed to sorbent surface protonation at acidic pH, resulting in the electrostatic repulsion of the positively charged Cr(III) species [58].

Response of the LS-based sensor to Cr(VI) at pH 7 corresponds to the theoretical response to a single-charged cation, although Cr(VI) is present at this pH as two anionic species, HCrO_4_^−^ and CrO_4_^2−^ [59]. At pH 2, the dominant Cr(VI) species are Cr_2_O_7_^2−^ and HCrO_4_^−^, with their ratio depending on the total Cr(VI) concentration [58]. The LS-based sensor response to Cr(VI) at pH 2 is about 45 mV/pX. A similar cationic response to Cr(VI) was observed for lignin-based sensors in the previous study [34] as well as for the chromate-selective sensor with a different type of membrane material—chalcogenide glass [60]. The sensitivity of both chalcogenide- and lignin-based sensors is pH-dependent, and, in the case of the previously studied lignin-based sensors, it was observed only at pH 2, while the LS-based sensor responded at both pH 7 and 2. It was hypothesized that the response mechanism of both chalcogenide glass and lignin-based electrodes is based on the combination of ion exchange and charge transfer, although no detailed mechanistic studies of the Cr(VI)-selective sensors’ response mechanism have been done.

Based on these findings, the LS-based sensor was expected to possess redox sensitivity, and the response of the sensor was evaluated in the solutions of two redox couples, ferro-ferricyanide Fe(CN)_6_^3^^−^^/4^^−^ and Cr(III)/Cr(VI), at pH 7 and pH 2. Response slopes were calculated by Equation (3):

(3) E=E0−RTzFlog(ab).

where *a* and *b* are the activities of the reduced and oxidized ions, respectively.

No redox response was obtained at pH 7; however, at pH 2, the sensor displayed response to both pairs (Figure 9). The sensor exhibited a near-theoretical response to the pair Fe(CN)_6_^3−/4−^, with a slope of −53.8 ± 3.2 mV, and a super-Nernstian response to the pair Cr(III)/Cr(VI), with a slope of −29.6 ± 8.3 mV.

Sensor responses in the individual solutions of ferrum and ferrocyanide at pH 7 and 2 are depicted in Figure 8 and Figure 9, respectively. The sensor response to Fe(CN)_6_^3−^ did not depend on pH and was ca. 19 mV/pX. The sensor displayed a similarly low response of ca. 17 mV/pX to Fe(CN)_6_^4−^ at pH 7, while at pH 2, a super-Nernstian anionic response of −72 mV/pX was observed. This response may be attributed to the combination of ion exchange and charge transfer processes involving the complexation of ferricyanide, with the functional groups of LS protonated at acidic pH and their subsequent oxidation.

With the aim to expand the applicability of lignin-based potentiometric sensors, the response of the LS-based PU sensor doped with 1% *w*/*w* MWCNTs towards ionic liquids (IL), 1-ethyl-3-methylimidazolium chloride ([C_2_mim]Cl) and choline chloride (ChCl), was examined (Figure S5, Supplementary Materials). These substances are widely used for different “green” syntheses and biomass fractionation purposes, whose presence in aquatic environments could be hazardous [9]. The measurements were carried out in Tris solution at pH 7. Although the sensor did detect both ILs, the sensitivity was relatively low, giving rise to response slopes of 12.0 ± 3.8 and 7.3 ± 0.4 mV for [C_2_mim]Cl and ChCl, respectively. The low response of the sensor may probably be due to steric hindrance from the structure of the ILs. However, further studies are required to evaluate the response mechanism of these potentiometric sensors to such types of compounds.

Since the LS-based sensor displayed a theoretical or even super-Nernstian response to several of the studied ions, the choice of the target ion, known as the primary ion, was not an obvious one for the determination of potentiometric selectivity. Taking into account the high and reproducible response to Cr(VI) and to facilitate a comparison with the previously obtained results for other types of lignin, this ion was considered the primary ion for the selectivity study. The matched potential method (MPM) was used for selectivity determination. Accordingly, the selectivity coefficient ($K_{A,B}^{\mathrm{pot}}$) is defined as the activity (concentration) ratio of the primary ion A (*a*_A_′ − *a*_A_) and the interfering ion B (*a*_B_), which give the same potential change in a reference solution (this one containing a fixed activity of primary ions *a*_A_) [57,61], and is expressed by Equation (4):

(4) $$K_{A,B}^{\mathrm{pot}}{=(a}_{A}^{\prime }{-a}_{A})/a_{B}$$

where $K_{A,B}^{\mathrm{pot}}$ is the selectivity coefficient, *a*_A_ and *a*_B_ are the activities of the primary ion A and the interfering ion B, respectively. The selectivity coefficient represents a numerical measure of the electrode membrane’s ability to discriminate the primary ion A (target ion) in the presence of the interfering ion B. A solution of 1.0 × 10^−4^ M of Cr (VI) in Tris 1 mM at pH 7 was used as the background. For the calculation of selectivity coefficients, ∆*E* = 20.0 mV was applied. The calculated values of selectivity coefficients are summarized in Table 1. The LS-based sensor displays low selectivity to Cr(VI) in the presence of most of the studied ions and is more selective to Cd(II) and especially to Ag(I) than to Cr(VI). However, in practice, selectivity to Ag(I) ions might not be necessary as they are rarely present in the analyzed samples.

These results show that this LS-based sensor displays sensitivity and low selectivity to several transition metal cations as well as a redox response. This behavior differs remarkably from the one observed for the other lignin-based sensors [5,33,34]. Sensors based on LignoBoost^®^ kraft lignin displayed a Nernstian response and high selectivity to Cu(II) and quite low or even no response to other cations except Hg(II), with which it interacted irreversibly, resulting in the loss of the electrode function [5]. Sensors based on kraft, organosolv, and lignosulphonate displayed sensitivity and selectivity to Cr(VI), redox sensitivity to the Cr(VI)/Cr(III) redox couple, and low sensitivity to transition metal cations such as Cu(II) and Pb(II), with higher responses observed for the LS-based sensor [34]. Furthermore, the three aforementioned sensors neither responded to nor were affected by the exposure to Hg(II). The reason for such disparity of the sensing properties can be attributed to the difference in chemical structure and composition between lignins obtained from different cooking processes, i.e., kraft lignin and LS, or isolation procedures, such as conventional kraft and LignoBoost^®^ kraft, and LS used previously and in this study [5,62]. LS contains phenolic groups with lower pKa than those in kraft lignin due to the strong electron-withdrawing moieties on the side chain (sulphonic groups at the benzylic carbon and conjugated double bonds) [63]. In addition to the sulphonic groups, the LS structures with phenolic hydroxyls have a high capacity for chelation of metal cations, thus acting as ionophores. This induces the formation of stable coordination complexes with a wide range of transition metal cations, influencing the sensing mechanism 

Other metal-sensitive sensors based on different polymeric membrane ion-selective electrodes incorporating ionophores, sensitive to different cations, have been widely reported in the literature [64,65,66,67,68,69,70,71,72,73,74,75,76,77,78,79,80,81,82,83,84,85,86,87,88]. Most of these sensors exhibit a near-Nernstian or Nernstian response to the different cations studied. The super-Nernstian behavior displayed by the LS-based PU membrane is probably due to the presence of strong anionic sites in the composite [89], e.g., inherent for the LS sulfonic acid groups. Hence, this work puts in evidence the decisive role of the lignin’s nature in the response of the corresponding potentiometric sensors.

The sensitivity of the LS-based polymeric sensor to redox potential and Cr(VI) was explained previously by the substructures with free phenolic hydroxyl groups involved in the eventual reversible hydroquinone/quinone type redox couples, favoring the complexation of Cr(VI) and its subsequent reduction to Cr(III) [34]. However, the high sensitivity of the LS-based sensor to several transition metals such as Zn, Cd, and Cu, found in the present work, was not registered in our previous study with another LS sample [34]. This behavior could be explained by the structural differences in LS samples induced by variations in eucalypt wood cooking conditions [35]. The LS sample used in this study was isolated from spent liquor after a more severe cooking process to produce dissolving pulp. The resulting LS was more condensed and richer in tannins (e.g., catechin, gallic and ellagic acids) than that used in the previous work [34]. Apparently, concomitant substructures, with two or three vicinal hydroxyl groups belonging to these polyphenolic concomitants, chelate easily with transition metal cations [90]. The effect of the presence of tannins on sensing properties can be corroborated by comparing the sensing behavior of polymers based on eucalyptus kraft lignins isolated by the conventional procedure and the LignoBoost^®^ process [5]. The latter had a higher content of total hydroxyl groups and a higher relative content of phenolic hydroxyl groups and concomitant tannins, which imparted to this material metal chelating properties; thus, the resulting sensor displayed high sensitivity to Cu(II) and no redox response. Indeed, the nature of the lignin used in the polymeric formulation of composites is decisive for the potentiometric sensor response, which is defined by the interplay between different functional groups and their concentrations.

### 3.4. DC Electrical Conductivity of LS-Based PU Polymer Membrane Composite Doped with Other Carbon Nanofillers

**Figure 10:**
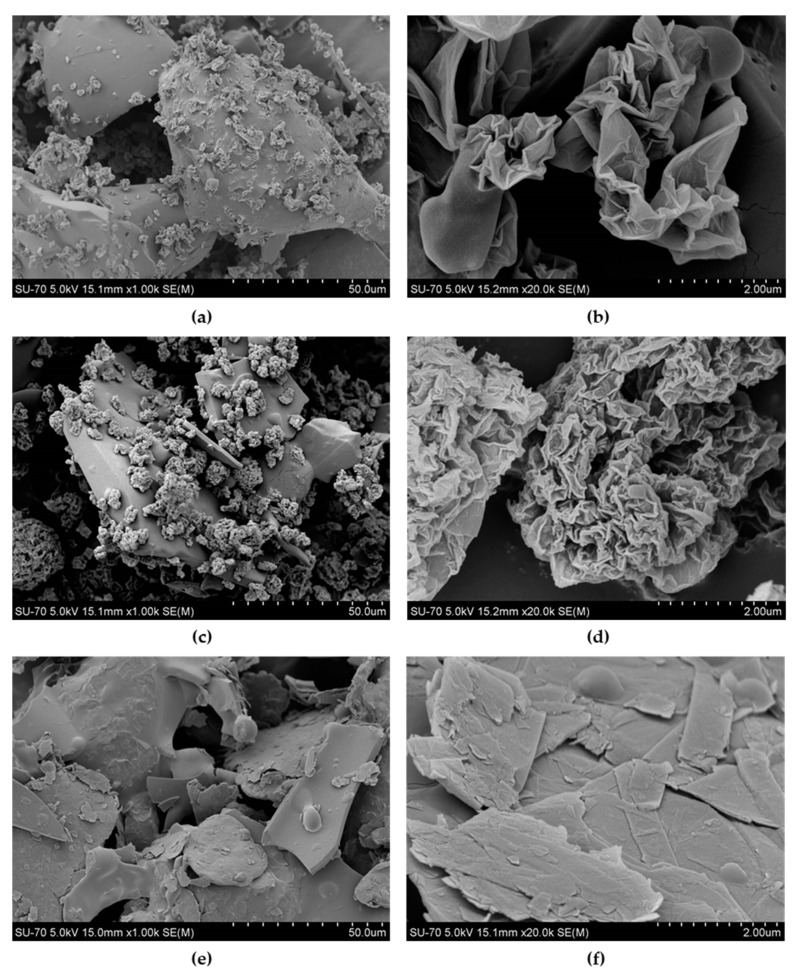
SEM images of the LS mixture with 1% *w*/*w* of GO (**a**,**b**), rGO (**c**,**d**), and Gr (**e**,**f**).

Considering the interesting sensing results obtained with MWCNTs as nanofillers in the composite with LS, the impact of other carbon nanofillers was evaluated, namely, graphene oxide (GO), reduced GO (rGO) and graphite (Gr), using similar LS-based PU formulations. The electrical conductivity of a composite occurs mostly as a result of the formation of a consistent network of nanofillers. SEM images of the LS mixture with 1% *w*/*w* GO, rGO, and Gr show different distribution patterns of nanofillers, as depicted in Figure 5 and Figure 10. As already mentioned, MWCNTs are highly entangled in a bundle structure with LS (Figure 5). In contrast, both GO and rGO are disoriented and mostly wrinkled and folded into a fuzzy structure (Figure 10). Finally, the graphite sheets are scattered randomly among LS particles (Figure 10). The entanglement and wrinkling of the nanofillers among LS particles may indicate that during the processing conditions, under mechanical stirring, these were not able to completely orient within the powder mixture [91]. This could explain the inappropriate distribution of nanofillers, failing to yield a conductive network within the composite matrix and affecting the electrical conductivity of the ensuing composites.

**Figure 11:**
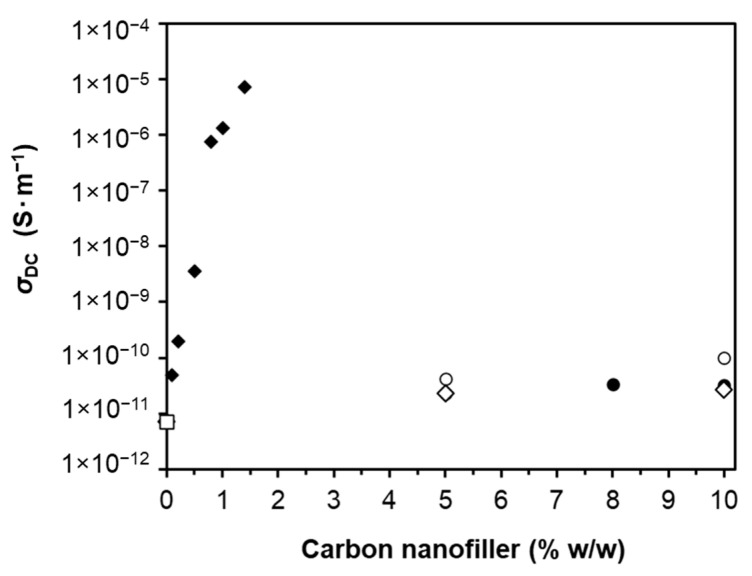
DC electrical conductivity, *σ*_DC_, at room temperature as a function of carbon nanofiller concentration (*υ*—MWCNT; ○—GO; ●—rGO; ◇—Gr; □—undoped) in the LS-based PU composite.

The impact of different carbon nanofillers with various concentrations on the DC electrical conductivity of LS-based PU polymer composites was examined, and the results are depicted in Figure 11. The highest MWCNT concentration of 1.4% *w*/*w* gave rise to almost 10^6^-fold higher electrical conductivity than the other nanofillers with the same concentration in the PU composite. Therefore, much larger amounts of GO, rGO, and Gr were required (up to 10% *w*/*w*) to achieve a visible increase in the conductivity of an LS-based PU composite material. The reason for this behavior is most probably due to the much higher aspect ratio of the MWCNT rods, which is more favorable to the creation of a continuous conductive network within the polymer matrix. Therefore, significantly improved electrical conductivity was obtained for smaller MWCNT loadings compared to the GO, rGO, and graphite samples at the same concentrations. In the case of the graphene sheets, the lack of conductivity improvement is probably due to the limited possibility of having GO, rGO, or graphite contact points and, thus, limited conduction [91].

## 4. Conclusions

Unlike PU composites based on kraft or organosolv lignins, MWCNT-doped LS-based PUs did not display a common percolation behavior due to the specificity of LS dissolution in the co-macromonomer used for the synthesis (PPGDI). Nevertheless, LS-based PUs doped with ≥ 1% *w*/*w* multiwall carbon nanotubes (MWCNTs) displayed relevant electrical conductivity suitable for sensor applications. Among examined carbon nanofillers within the range of 0–10% *w*/*w* (multiwall nanotubes, graphene oxides, and graphite), only MWCNTs provided significantly improved electrical conductivity to LS-based PU films suitable for sensing applications. Applied as a potentiometric sensor, MWCNT-doped LS-based PUs showed sensitivity to Cu(II), Zn(II), Cd(II), Cr(III), Cr(VI), Hg(II), and Ag(I) at pH 7 and exhibited a response to the Cr(VI)/Cr(III) redox pair at pH 2. The highest selectivity in solution at pH 7 was observed for Cd(II) and especially for Ag(I) ions. It is suggested that LS obtained from the same wood but under different cooking conditions and containing polyphenolic concomitants can exhibit distinct sensing performance due to the presence of corresponding structural units with chelating vicinal phenolic hydroxyls. The LS-based conducting polymer revealed a weak but not negligible response toward ionic liquids, [C_2_mim]Cl and ChCl. An additional study is necessary to find the sensitivity of the obtained sensor membranes to different classes of potentially hazardous compounds for their reliable detection in solutions.

## Figures and Tables

**Table 1:** Selectivity coefficients, $K_{A,B}^{\mathrm{pot}}$, of the LS-based sensor towards Cr(VI), determined using the matched potential method (mean values of at least three values).

Interferent Ion	$K_{A,B}^{pot}$
Zn(II)	0.29 ± 0.08
Cd(II)	1.49 ± 0.41
Cu(II)	0.40 ± 0.15
Cr(III)	0.53 ± 0.28
Hg(II)	0.54 ± 0.11
Ag(I)	2.66 ± 0.62
Fe(III)	0.50 ± 0.26
Fe(II)	0.65 ± 0.14

## Data Availability

Data sharing not applicable.

## Supplementary Materials

The following are available online at https://www.mdpi.com/article/10.3390/ma14185331/s1, Figure S1: TEM image of the LS-based PU film doped with 1% *w*/*w* MWCNTs, Figure S2: SEM images of the cross-section of LS-based PU films, Figure S3: −Log *A* versus *s* and log *A*/*s* versus T plots of LS-based PU film doped with 1% *w*/*w* of MWCNTs, Figure S4: Calibration curves of the LS-based PU composite membrane sensor doped with 1% *w*/*w* MWCNTs towards four selected cations, Figure S5: Chemical structure of the ILs studied: (a) 1-ethyl-3-methylimidazolium chloride and (b) choline chloride. Table S1: Assignment of bands in FTIR-ATR spectra of purified LS from eucalyptus thick sulfite pulping liquor and LS-based PUs undoped (LS-PU) and doped with 1% *w*/*w* MWCNTs (LS-PU-CNT), Table S2: Sensitivity characteristics of the LS-based PU membrane sensor doped with 1% *w*/*w* MWCNTs at pH 7.
