# Supplementary material for: Lignosulfonate-Based Conducting Flexible Polymeric Membranes for Liquid Sensing Applications
Source: Materials (Basel). 2021 Sep 15;14(18):5331. doi: 10.3390/ma14185331 (PMC8470934; doi:10.3390/ma14185331)
Supplement: Supplementary file 1 [file materials-14-05331-s001.zip › materials-1342504-supplementary.pdf]

Supplementary Materials

Lignosulfonate-Based Conducting Flexible Polymeric Membranes for Liquid Sensing Applications

Sandra Magina [1], Alisa Rudnitskaya [2], Silvia Soreto [3], Luís Cadillon Costa [3], Ana Barros-Timmons [1] and Dmitry V. Evtuguin [1,*]

[1] CICECO—Aveiro Institute of Materials and Department of Chemistry, University of Aveiro, Aveiro, 3810-193, Portugal; smagina@ua.pt (S.M.); anabarros@ua.pt (A.B.-T.)
[2] CESAM and Department of Chemistry, University of Aveiro, 3810-193 Aveiro, Portugal; alisa@ua.pt
[3] I3N and Department of Physics, University of Aveiro, 3810-193 Aveiro, Portugal; silvia.soreto@ua.pt (S.S.); kady@ua.pt (L.C.C.)
* Correspondence: dmitrye@ua.pt

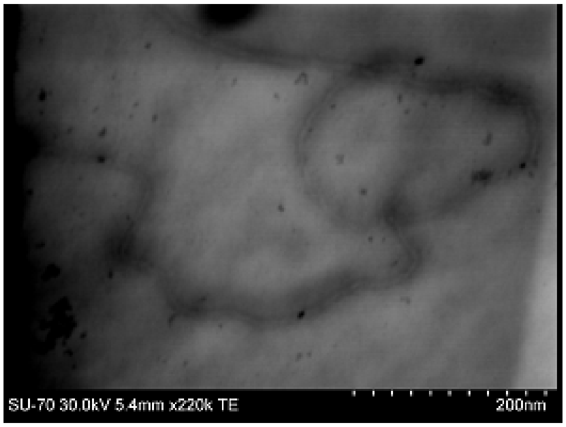

Figure S1. TEM image of the LS-based PU film doped with 1% w/w MWCNTs.

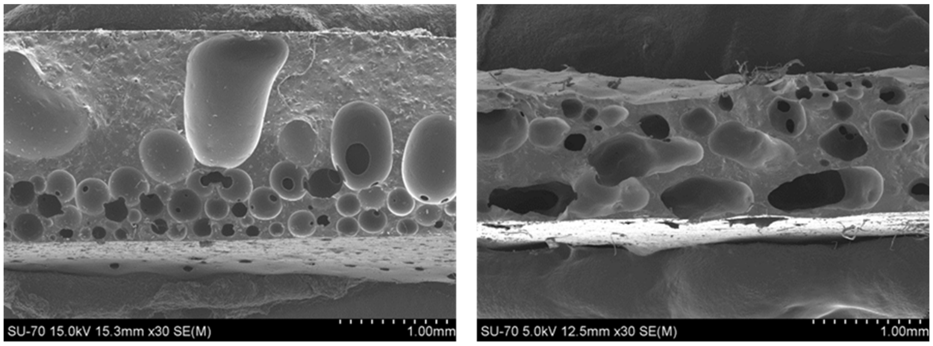

(a) (b)

Figure S2. SEM images of the cross-section of LS-based PU films (a) undoped and (b) doped with 1% w/w of MWCNTs.

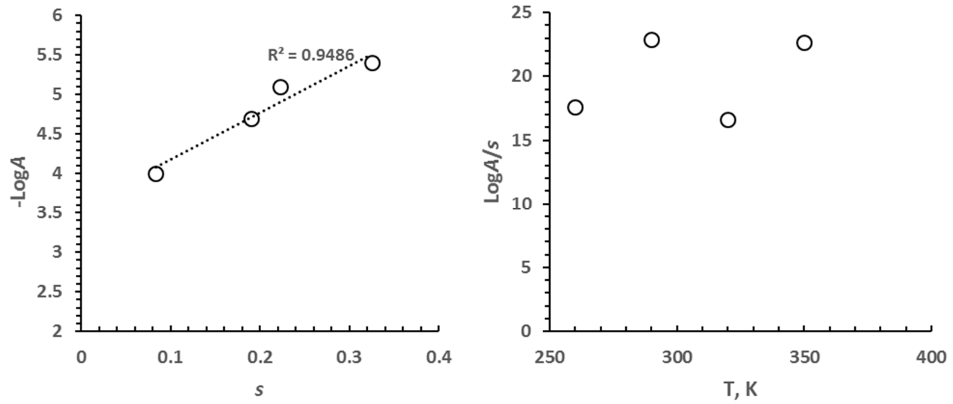

Figure S3. -Log*A* versus *s* and Log*A*/*s* versus *T* plots of LS-based PU film doped with 1% w/w of MWCNTs.

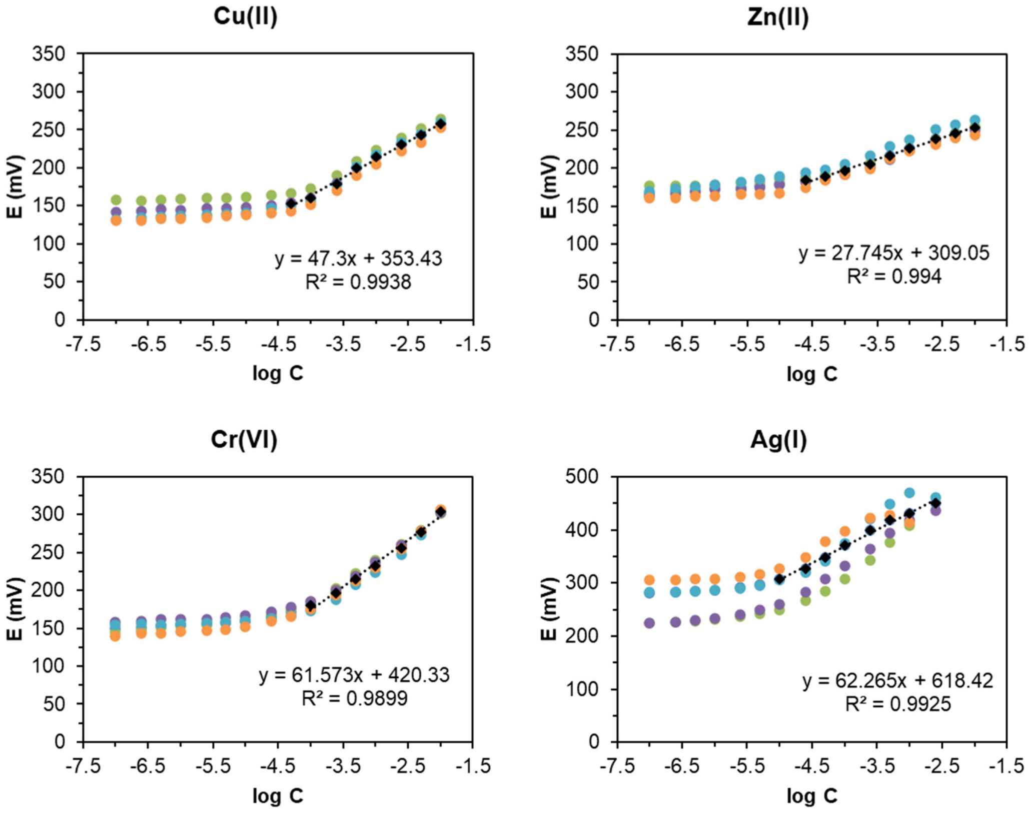

Figure S4. -Calibration curves LS-based PU composite membrane sensor doped with 1% w/w MWCNTs towards four selected cations (four calibration curves – coloured dots – for each cation and the re-spective average response slope with linear trendline – black).

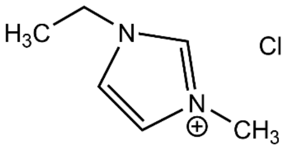

(**a**)

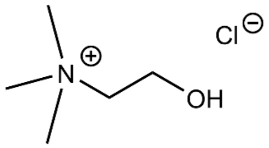

(**b**)

Figure S5. Chemical structure of the ILs studied: (a) 1-ethyl-3-methylimidazolium chloride and (b) choline chlorid**e.**

Table S1. Assignment of bands in FTIR-ATR spectra of purified LS from eucalypt thick sulphite pulping liquor and LS-based PU undoped (LS-PU) and doped with 1% w/w MWCNTs (LS-PU-CNT) [1,2,11,3–10].

**LS (cm^{-1})**	**LS-PU/ LS-PU-CNT (cm^{-1})**	**Assignment**
3356	3298	O-H stretching, H-bonded
2940/2848	2966/2916/ 2860	C-H stretching in methyl, methylene and O-CH_3 groups
-	1722	C=O stretching (urethane group)
1604		Aryl ring stretching, symmetric
-	1530	N-H bending (secondary amine in urethane group)
1510		Aryl ring stretching, asymmetric
1460		C-H bending in O-CH_3 groups, asymmetric
1426		Aromatic skeletal vibration combined with C-H bending in O-CH_3 groups, asymmetric in-plane
1330		C_{aryl}-O vibrations, SO_3H groups (S=O stretching vibration)
-	1220	C-N stretching (urethane group)
1210		C_{aryl}-O vibrations, C-C, C-O, C=O stretching, metallic salt of SO_3H groups (S=O stretching vibration)
1154		Aromatic C-H in-plane deformation, SO_3H groups
1112		Aromatic C-H in-plane deformation, C-O-C groups, metallic salt of SO_3H groups
-	1086	C-O-C vibration (polyether bridges)
1034		Aromatic C-H in-plane deformation related with C-O, C-C stretching and C-OH stretching, SO_3H groups, C_{alkyl}-O ether vibrations (O-CH_3 and β–O-4)
914/818		C-H deformation out-of-plane, aromatic ring
650/630		SO_3H groups (S-O stretching vibration/C-S stretching vibration)
630		SO_3H groups (C-S stretching vibration)

Table S2. Sensitivity characteristics of LS-based PU membrane sensor doped with 1% *w/w* MWCNTs at pH 7.

Cation	Slope (mV/decade)	Detection limit (M)	Linear range (M)
Cu(II)	47.3 ± 2.1	4.6×10^{-5}	5.0×10^{-5}–1×10^{-2}
Cd(II)	20.3 ± 2.5	4.2×10^{-6}	5.0×10^{-6}–1×10^{-2}
Zn(II)	27.2 ± 0.7	1.2×10^{-5}	1.5×10^{-5}–1×10^{-2}
Hg(II)	40.9 ± 5.7	3.3×10^{-5}	5.0×10^{-5}–1×10^{-2}
Cr(VI)	61.6 ± 3.6	6.1×10^{-5}	1.0×10^{-4}–1×10^{-2}
Cr(III)	68.3 ± 1.8	6.2×10^{-5}	1.0×10^{-4}–1×10^{-2}
Ag(I)	62.1 ± 15.5	4.9×10^{-6}	1.0×10^{-5}–2.2×10^{-3}

References

1. Agarwal, U.P.; Atalla, R.H. Vibrational Spectroscopy. In *Lignin and lignans: advances in chemistry*; Heitner, C., Dimmel, D., Schmidt, J.A., Eds.; Taylor and Francis Group, LLC: Boca Raton, FL, 2010; pp. 103–136 ISBN 9781574444865.
2. Boeriu, C.G.; Bravo, D.; Gosselink, R.J.A.; van Dam, J.E.G. Characterisation of structure-dependent functional properties of lignin with infrared spectroscopy. *Ind. Crops Prod.* **2004**, *20*, 205–218, doi:10.1016/j.indcrop.2004.04.022.
3. Dawy, M.; Shabaka, A.A.; Nada, A.M.A. Molecular structure and dielectric properties of some treated lignins. *Polym. Degrad. Stab.* **1998**, *62*, 455–462, doi:10.1016/S0141-3910(98)00026-3.
4. Collier, W.; Kalasinsky, V.F.; Schultz, T.P. Infrared study of lignin: Assignment of methoxyl C-H bending and stretching bands. *Holzforschung* **1997**, *51*, 167–168, doi:10.1515/hfsg.1997.51.2.167.
5. Bellamy, L.J. *The Infrared Spectra of Complex Molecules. Volume 2. Advances in Infrared group frequencies*; 2nd Ed.; Chapmanand Hall: London and New York, 1980; ISBN 978-94-011-6522-8.
6. Collier, W.E.; Schultz, T.P.; Kalasinsky, V.F. Infrared study of lignin: reexamination of acryl-alkyl ether C-O stretching. *Holzforschung* **1992**, *46*, 523–528.
7. Detoni, S.; Hadzi, D. Infra-red spectra of some organic sulphur-oxygen compounds. *Spectrochim. Acta* **1956**, *11*, 601–608, doi:10.1016/S0371-1951(56)80102-1.
8. Marques, A.P.; Evtuguin, D. V.; Magina, S.; Amado, F.M.L.; Prates, A. Chemical Composition of Spent Liquors from Acidic Magnesium–Based Sulphite Pulping of Eucalyptus globulus. *J. Wood Chem. Technol.* **2009**, *29*, 322–336, doi:10.1080/02773810903207754.
9. Hergert, H.L. Infrared spectra. In *Lignins: Occurrence, formation, structure and reactions*; Sarkanen, K. V., Ludwig, C.H., Eds.; John Wiley & Sons, Inc.: New York, 1971; pp. 267–297.
10. Faria, F.A.C.; Evtuguin, D. V.; Rudnitskaya, A.; Gomes, M.T.S.R.; Oliveira, J.A.B.P.; Graça, M.P.F.; Costa, L.C. Lignin-based polyurethane doped with carbon nanotubes for sensor applications. *Polym. Int.* **2012**, *61*, 788–794, doi:10.1002/pi.4140.
11. Zou, J.; Chen, Y.; Liang, M.; Zou, H. Effect of hard segments on the thermal and mechanical properties of water blown semi-rigid polyurethane foams. *J. Polym. Res.* **2015**, *22*, doi:10.1007/s10965-015-0770-y.
